# A study of genomic diversity in populations of Maharashtra, India, inferred from 20 autosomal STR markers

**DOI:** 10.1186/s13104-021-05485-z

**Published:** 2021-02-23

**Authors:** Ashish Badiye, Neeti Kapoor, R. K. Kumawat, Shivani Dixit, Aditi Mishra, Akansha Dixit, Prachi Kathane, Sudeshna Bag, Vaishnavi Thakre, Kamlesh Kaitholia, Ankit Srivastava, Gyaneshwer Chaubey, Pankaj Shrivastava

**Affiliations:** 1 Department of Forensic Science, Government Institute of Forensic Science, Nagpur, Maharashtra India; 2 DNA Division, State Forensic Science Laboratory, Jaipur, Rajasthan India; 3 DNA Fingerprinting Unit, State Forensic Science Laboratory, Sagar, M.P. 470001 India; 4 Dr. A.P.J. Abdul Kalam Institute of Forensic Science & Criminology, Bundelkhand University, Jhansi, U.P. 284128 India; 5 Cytogenetics Laboratory, Dept of Zoology, Banaras Hindu University, Varanasi, India

**Keywords:** STRs, Heterozygosity, Power of discrimination, Power of exclusion, Maharashtra

## Abstract

**Objective:**

This study was planned to evaluate the genetic diversity in the admixed and *Teli* (a Hindu caste) populations of Maharashtra, India using 20 autosomal Short Tandem Repeat (STR) genetic markers. We further investigated the genetic relatedness of the studied populations with other Indian populations.

**Results:**

The studied populations showed a wide range of observed heterozygosity viz. 0.690 to 0.918 for the admixed population and 0.696 to 0.942 for the Teli population. This might be due to the multi-directional gene flow. The admixed and Teli populations also showed a high degree polymorphism which ranged from 0.652 to 0.903 and 0.644 to 0.902, respectively. Their combined value of matching probability for all the studied loci was 4.29 × 10^–25^ and 5.01 × 10^–24^, respectively. The results of Neighbor-Joining tree and Principal Component Analysis showed that the studied populations clustered with the general populations of Jharkhand, UttarPradesh, Rajasthan and Central Indian States, as well as with the specific populations of Maharashtra (*Konkanastha Brahmins*) and Tamil Nadu (*Kurmans*). Overall, the obtained data showed a high degree of forensic efficacy and would be useful for forensic applications as well as genealogical studies.

## Introduction

**Fig. 1:**
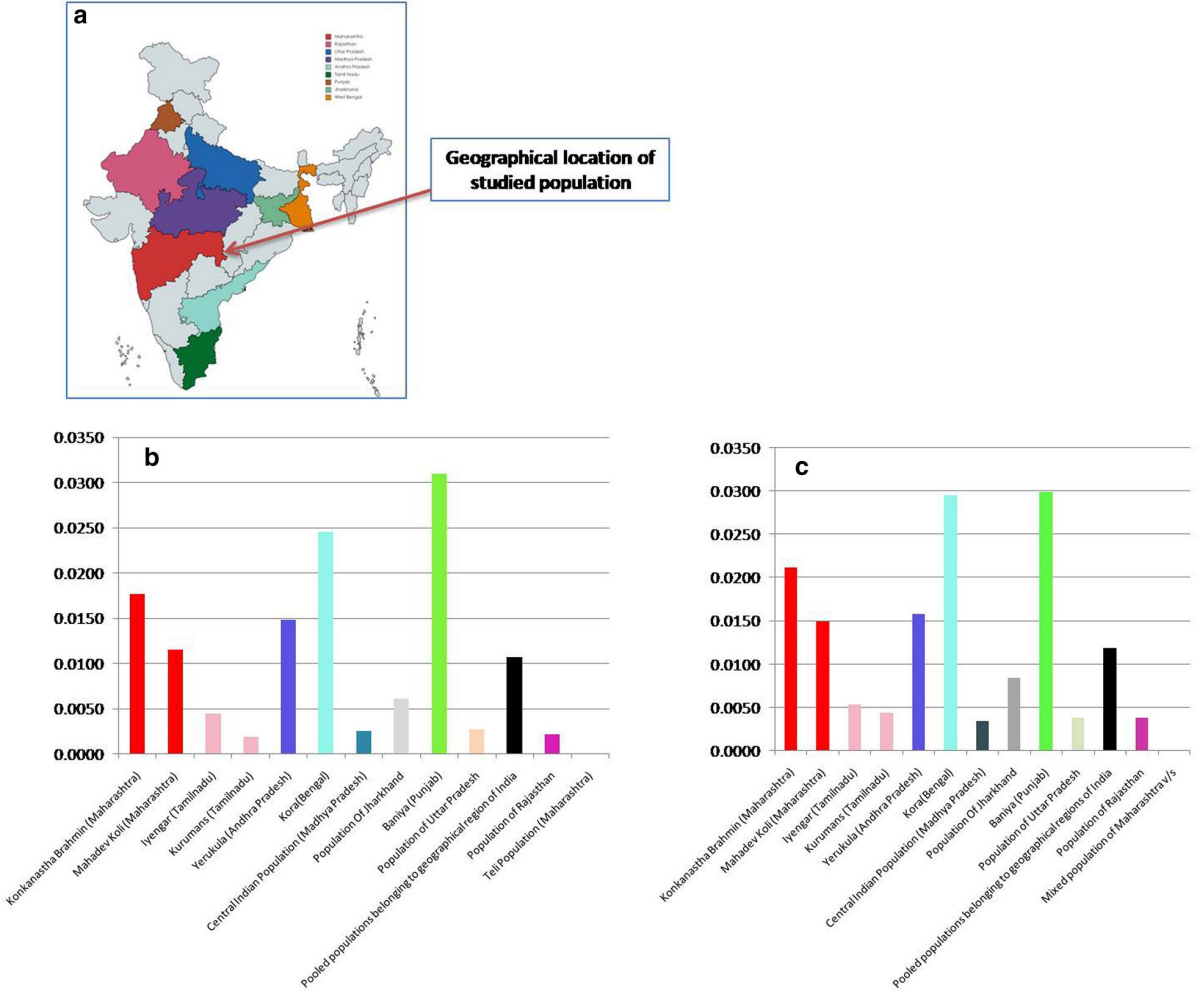
**a** Geographical locations of the studied and compared populations (map is not under copyright; map was created with mapchart.net). **b** Phylogenetic distance between studied admixed population of Maharashtra and compared populations. **c** Phylogenetic distance between studied *Teli* population of Maharashtra and compared populations

The state of Maharashtra is located in the western peninsular region of India. It is the third-largest state by area and the second-most populous state in the country. It shares its geographical boundaries with the states of Karnataka and Goa in the South, Telangana in the South-east, Chhattisgarh in the East, Gujarat and Madhya Pradesh in the North, Dadra-Nagar Haveli in the North-west, and the Arabian Sea in the West (Fig. 1). As per the 2011 census, Maharashtra has a population 112,374,333, which contributes to9.28% of the total Indian population[1]. Although '*Marathi*' is the native and official language of the state, several regional languages and their dialects are also spoken across Maharashtra, because people from different regions such as Biharis, Gujaratis, Sindhis, Punjabis, Parsis, Marwaris, Kannadas and Tamilians are settled across the state [2].The population of Maharashtra is so diverse because it served as a geographical margin between Ancestral North India (ANI) and Ancestral South India (ASI) [3, 4], and has witnessed several migration waves over centuries. Interaction between these populations over innumerable generations have subsequently influenced the genomic diversity of the state [3, 5–7]. Not only people from varied regions, but people of different castes also reside in Maharashtra(Hindu hierarchical groups) like *Teli* caste. The '*Teli*' community derives its name from *Sanskrit* word '*talika*' or '*taila*' which means oil and it indicates towards the traditional occupation of the *Teli* community which was to extract oil from sesame and mustard seeds. One of the Hindu mythological references of the *Teli* caste indicates that the first *Teli* individual was created by 'Lord Shiva' to rub him with oil [8].

The overall social, cultural, and lingual diversity of the state of Maharashtra led us to evaluate the genomic diversity of the admixed and *Teli* populations of this state. The genomic data of the selected populations was evaluated using in-silico or computational techniques through various population data software and servers such as GeneMapper™ ID-X, Arlequin v3.5, POPTREE2, PAST 3.02a, etc. The in-silico techniques have served as an efficient approach for the evaluation of very large genomic data sets such as STRs, SNPs, large sequence and NGS data [9–12] because they could quickly analyze large data sets with high-throughput and accuracy.

## Main text

To investigate the genetic diversity of the admixed and *Teli* population of Maharashtra, we randomly selected 158 and 69 unrelated healthy adults, respectively.The subjects in the admixed group belonged to almost all the population groups residing in the state of Maharashtra and hence represented the diverse population of Maharashtra. On the contrary, the subjects in the *Teli* group were recruited only from the*Teli* community. An online randomization tool-the randomizer (www.random.org) was used to randomly allocate subjects to each group, prior to the sample collection.

First, an interview was conducted to confirm that each participant’s ancestors have been residing within the geographical boundaries of Maharashtra for more than three generations. Next, blood samples were collected from each participant following the ethical guidelines and the declaration of Helsinki [13]. The collected blood samples were subjected to the Phenol–Chloroform Isoamyl Alcohol (PCIA) organic extraction method for DNA extraction[14]. The extracted DNA was quantified using the PowerQuant® DNAQuantification kit (Promega, Madisson, USA-Promega) in a Real-Time Polymerase Chain Reaction machine (RT-PCR-7500) (Thermo Fisher Scientific, CA, USA) as recommended by the manufacturer(except for the half-reaction volume). A 500 pg DNA template was used to amplify 21 autosomal STR loci using PowerPlex® 21 System (Promega) on Veriti™ 96-Well Fast Thermal Cycler (ThermoFisher Scientific, CA, USA) as per manufacturer’s recommendations(except for the half-reaction volume). The amplified DNA fragments were separated by capillary electrophoresis using POP™-4, 36 cm capillary array and Genetic Analyzer 3500XL (Thermo Fisher Scientific, CA, USA) as recommended by the manufacturer. The allelic ladder provided with the kit was used for the allocation of the allele number at the particular loci. The DNA profile was evaluated using the GeneMapper^TM^ID-X v1.5 software (Thermo Fisher Scientific, CA, USA). Positive and negative controls were used in the experiment to assure the quality control. Additionally, the authors conducting this study have passed the proficiency test conducted by GITAD, Spain (http://gitad.ugr.es/principal.htm).

The obtained genetic data was analyzed using statistical software.The GenAlex 6.5 software [15] was used to calculate the allele frequencies and the PowerStats v1.2 spreadsheet program [16] was used to calculate various forensic parameters namely polymorphic information content (PIC), power of discrimination (PD), power of exclusion (PE), matching probability (PM) and paternity index (PI). The observed heterozygosity (Hobs), expected heterozygosity (Hexp) and Hardy–Weinberg equilibrium (HWE) were calculated using the Arlequin v3.5 software [17]. POPTREE2 program [18] was used to draw neighborjoining (NJ) tree and Nei's genetic distances [19] among the compared populations. The PAST 3.02a software [20] was used for the graphical representation of genetic distances among the compared populations, based on the Principal component analysis (PCA). Maximum likelihood (ML) phylogenetic tree was reconstructed as described earlier [21].

A total of 228 alleles, with an average of 11.4 alleles per locus were observed for the admixed population group, while a total of 194 alleles, with an average of 9.7 alleles per locus were observed for the *Teli* population group. The locus D3S1358 showed minimum allele number of 5, and loci Penta E and D21S11 showed maximum allele number of 19 in the admixed population group. On the other hand, in the *Teli* population group, the loci D3S1358 and TPOX showed minimum allele number of 5, and locus Penta E showed maximum allele number of 17. The range of allele frequencies for the admixed and *Teli* population group were 0.003 to 0.427 and 0.007 to 0.435, respectively. Allele 11 of locus TPOX was observed to be the most frequent allele in both admixed (Table 1) and *Teli* (Additional file 1: Table S1) population groups. All the studied loci for both population groups followed the Hardy–Weinberg equilibrium after applying Bonferroni correction (P = 0.05/20, at a 95% significance level).

**Table 1:** Allele frequencies and forensic parameters for the 20 autosomal STR loci intheadmixed population of Maharashtra, India (n = 158)

Allele	D3S1358	D1S1656	D6S1043	D13S317	Penta E	D16S539	D18S51	D2S1338	CSF1PO	Penta D	TH01	vWA	D21S11	D7S820	D5S818	TPOX	D8S1179	D12S391	D19S433	FGA
5	–	–	–	–	0.054	–	–	–	–	–	–	–	–	–	–	–	–	–	–	–
6	–	–	–	–	–	–	–	–	–	0.003	0.275	–	–	–	–	–	–	–	–	–
7	–	–	–	0.019	0.060	–	–	–	–	0.009	0.111	–	–	0.032	–	0.003	–	–	–	–
8	–	0.044	–	0.177	0.009	0.044	–	–	–	0.009	0.155	–	–	0.250	–	0.288	0.009	–	–	–
9	–	0.003	0.009	0.127	0.013	0.149	–	–	0.009	0.209	0.310	–	–	0.073	0.032	0.155	–	–	–	–
9.3	–	–	–	–	–	–	–	–	–	–	0.142	–	–	–	–	–	–	–	–	–
10	–	0.006	0.003	0.073	0.038	0.114	0.009	–	0.203	0.256	0.006	0.003	–	0.196	0.136	0.095	0.177	–	–	–
10.3	–	–	0.009	–	–	–	–	–	–	–	–	–	–	–	–	–	–	–	–	–
10.4	–	–	–	–	–	–	–	–	–	0.003	–	–	–	–	–	–	–	–	–	–
11	–	0.165	0.272	0.244	0.177	0.307	0.025	–	0.335	0.269	–	–	–	0.253	0.316	0.427	0.114	–	0.006	–
11.3	–	–	0.009	–	–	–	–	–	–	–	–	–	–	–	–	–	–	–	–	–
12	–	0.060	0.206	0.275	0.092	0.206	0.079	–	0.364	0.117	–	–	–	0.155	0.345	0.028	0.044	–	0.076	–
12.2	–	–	–	–	–	–	–	–	–	–	–	–	–	–	–	–	–	–	0.016	–
12.3	–	–	0.003	–	–	–	–	–	–	–	–	–	–	–	–	–	–	–	0.006	–
13	–	0.139	0.120	0.060	0.051	0.165	0.149	–	0.076	0.076	–	0.003	–	0.032	0.165	0.003	0.168	–	0.320	–
13.2	–	–	–	–	–	–	–	–	–	–	–	–	–	–	–	–	–	–	0.022	–
13.3	–	–	–	–	–	–	–	–	–	–	–	–	–	–	–	–	–	–	0.028	–
14	0.073	0.098	0.066	0.025	0.060	0.016	0.259	–	0.009	0.038	–	0.136	–	0.009	0.006	–	0.209	–	0.184	–
14.2	–	–	–	–	–	–	–	–	–	–	–	–	–	–	–	–	–	–	0.070	–
14.3	–	–	–	–	–	–	–	–	–	–	–	–	–	–	–	–	–	–	0.003	–
15	0.335	0.136	0.006	–	0.089	–	0.184	––	0.003	0.009	–	0.133	–	–	–	–	0.171	0.009	0.120	–
15.2	–	–	–	–	–	–	–	–	–	–	–	–	–	–	–	–	–	–	0.054	–
15.3	–	0.022	–	–	–	–	–	–	–	–	–	–	–	–	–	–	–	–	–	–
16	0.269	0.152	0.003	–	0.079	–	0.104	0.003	–	–	–	0.203	–	–	–	–	0.089	0.009	0.054	0.003
16.2	–	–	–	–	–	–	–	–	–	–	–	–	–	–	–	–	–	–	0.028	–
16.3	–	0.041	–	–	–	–	–	–	–	–	–	–	–	–	–	–	–	–	–	–
16.4	–	–	–	–	0.003	–	–	–	–	–	–	–	–	–	–	–	–	–	–	–
17	0.228	0.089	0.044	–	0.095	–	0.066	0.022	–	–	–	0.285	–	–	–	–	0.019	0.133	0.013	–
17.3	–	0.019	–	–	–	–	–	–	–	–	–	–	–	–	–	–	–	0.009	–	–
18	0.095	0.013	0.111	–	0.108	–	0.054	0.180	–	–	–	0.190	–	–	–	–	–	0.259	–	0.006
18.3	–	0.006	–	–	–	–	–	–	–	–	–	–	–	–	–	–	–	0.016	–	–
19	–	–	0.073	–	0.025	–	0.038	0.158	–	–	–	0.044	–	–	–	–	–	0.149	–	0.079
19.2	–	–	–	–	–	–	–	–	–	–	–	–	–	–	–	–	–	0.003	–	0.006
19.3	–	0.006	–	–	–	–	–	–	–	–	–	–	–	–	–	–	–	0.003	–	–
20	–	–	0.060	–	0.028	–	0.016	0.133	–	–	–	0.003	–	–	–	–	–	0.127	–	0.092
20.2	–	–	–	–	–	–	–	–	–	–	–	–	–	–	–	–	–	–	–	0.003
21	–	–	0.003	–	0.013	–	0.013	0.038	–	–	–	–	–	–	–	–	–	0.101	–	0.117
21.2	–	–	–	–	–	–	–	–	–	–	–	–	–	–	–	–	–	–	–	0.006
22	–	–	–	–	0.003	–	–	0.076	–	–	–	–	–	–	–	–	–	0.082	–	0.142
22.2	–	–	–	–	–	–	–	–	–	–	–	–	–	–	–	–	–	–	–	0.003
23	–	–	–	–	0.003	–	–	0.206	–	–	–	–	–	–	–	–	–	0.041	–	0.155
23.2	–	–	–	–	–	–	–	–	–	–	–	–	–	–	–	–	–	–	–	0.009
24	–	–	–	–	–	–	–	0.095	–	–	–	–	–	–	–	–	–	0.032	–	0.146
24.2	–	–	–	–	–	–	–	–	–	–	–	–	–	–	–	–	–	–	–	0.006
25	–	–	–	–	–	–	–	0.070	–	–	–	–	–	–	–	–	–	0.013	–	0.158
25.2	–	–	–	–	–	–	–	–	–	–	–	–	–	–	–	–	–	–	–	0.006
26	–	–	–	–	–	–	–	0.013	–	–	–	–	–	–	–	–	–	0.013	–	0.057
27	–	–	–	–	–	–	0.003	0.003	–	–	–	–	0.006	–	–	–	–	–	–	0.003
27.3	–	–	–	–	–	–	–	–	–	–	–	–	0.006	–	–	–	–	–	–	–
28	–	–	–	–	–	–	–	–	–	–	–	–	0.139	–	–	–	–	–	–	–
28.3	–	–	–	–	–	–	–	–	–	–	–	–	0.028	–	–	–	–	–	–	–
29	–	–	–	–	–	–	–	0.003	–	–	–	–	0.184	–	–	–	–	–	–	–
29.2	–	–	–	–	–	–	–	–	–	–	–	–	0.003	–	–	–	–	–	–	–
29.3	–	–	–	–	–	–	–	–	–	–	–	–	0.009	–	–	–	–	–	–	–
30	–	–	–	–	–	–	–	–	–	–	–	–	0.215	–	–	–	–	–	–	–
30.2	–	–	–	–	–	–	–	–	–	–	–	–	0.016	–	–	–	–	–	–	–
31	–	–	–	–	–	–	–	–	–	–	–	–	0.035	–	–	–	–	–	–	–
31.1	–	–	–	–	–	–	–	–	–	–	–	–	0.006	–	–	–	–	–	–	–
31.2	–	–	–	–	–	–	–	–	–	–	–	–	0.082	–	–	–	–	–	–	–
31.3	–	–	–	–	–	–	–	–	–	–	–	–	0.003	–	–	–	–	–	–	–
32	–	–	–	–	–	–	–	–	–	–	–	–	0.003	–	–	–	–	–	–	–
32.1	–	–	–	–	–	–	–	–	–	–	–	–	0.006	–	–	–	–	–	–	–
32.2	–	–	–	–	–	–	–	–	–	–	–	–	0.155	–	–	–	–	–	–	–
32.3	–	–	–	–	–	–	–	–	–	–	–	–	0.003	–	–	–	–	–	–	–
33.2	–	–	–	–	–	–	–	–	–	–	–	–	0.089	–	–	–	–	–	–	–
34.2	–	–	–	–	–	–	–	–	–	–	–	–	0.009	–	–	–	–	–	–	–
Pm	0.104	0.030	0.050	0.069	0.020	0.070	0.046	0.042	0.136	0.079	0.090	0.067	0.046	0.080	0.129	0.143	0.050	0.040	0.057	0.030
PIC	0.707	0.875	0.824	0.781	0.903	0.771	0.834	0.845	0.655	0.768	0.735	0.775	0.844	0.775	0.689	0.652	0.825	0.841	0.814	0.865
Hexp	0.749	0.886	0.841	0.807	0.909	0.799	0.850	0.861	0.708	0.797	0.771	0.804	0.859	0.803	0.734	0.701	0.844	0.856	0.831	0.877
Hobs	0.715	0.918	0.816	0.797	0.854	0.797	0.848	0.899	0.690	0.835	0.791	0.778	0.867	0.835	0.747	0.734	0.854	0.797	0.778	0.861
P–value	0.489	0.473	0.080	0.489	0.221	0.708	0.233	0.439	0.793	0.786	0.402	0.852	0.000	0.051	0.080	0.061	0.377	0.051	0.000	0.770
PI	3.038	3.292	2.257	2.724	3.435	2.469	3.435	1.612	1.756	1.881	2.469	3.591	4.938	3.762	2.394	2.257	3.038	6.077	2.469	1.975
PE	0.452	0.832	0.630	0.594	0.704	0.594	0.691	0.793	0.413	0.666	0.583	0.560	0.729	0.666	0.504	0.483	0.704	0.594	0.560	0.716
PD	0.896	0.970	0.950	0.931	0.980	0.930	0.954	0.958	0.864	0.921	0.910	0.933	0.954	0.920	0.871	0.857	0.950	0.960	0.943	0.970

*Pm* matching probability, *PIC* polymorphic information content, *Hexp* expected heterozygosity,*Hobs* observed heterozygosity, *P value* HWE test, *PI* paternity index, *PE* Power of exclusion, *PD* power of discrimination

The obtained forensic efficacy parameters for the admixed and *Teli* populations of Maharashtra are shown in Table 1 and Additional file 1: Table S1, respectively. The locus Penta E was the most polymorphic loci in both the population groups, with a value of 0.903 in the admixed and 0.902 in the *Teli* population group. In contrast, locus TPOX was the least polymorphic among all the studied loci, with a value of 0.652 in the admixed and 0.644 in the *Teli* population group. A high range of observed heterozygosity (Hobs) value in the admixed (0.690 to 0.918) group as well as the *Teli* (0.696 to 0.942) group might have resulted from the inflow of genes in the studied populations from various directions.

The power of discrimination (PD) for the admixed population group ranged from 0.857 (TPOX) to 0.980 (Penta E) and the PD for the *Teli* population group ranged from 0.849 (TPOX) to 0.974 (Penta E), with the combined value for all the studied loci as 1, for both the groups. In the admixed group, the power of exclusion (PE) ranged from 0.413 (CSF1PO) to 0.832 (D1S1656) with the combined value for all the studied loci as 0.999999998666, whereas in the *Teli* group,the PE range was 0.422 (D5S818) to 0.882 (D1S1656) with the combined value for all the studied loci as 0.999999999652. The combined value of matching probability for all the studied 20 autosomal STR loci was found to be 4.29 × 10^–25^ (Table 1) for the admixed group and 5.01 × 10^–24^ (Additional file 1: Table S1) for the *Teli* group.

**Fig. 2:**
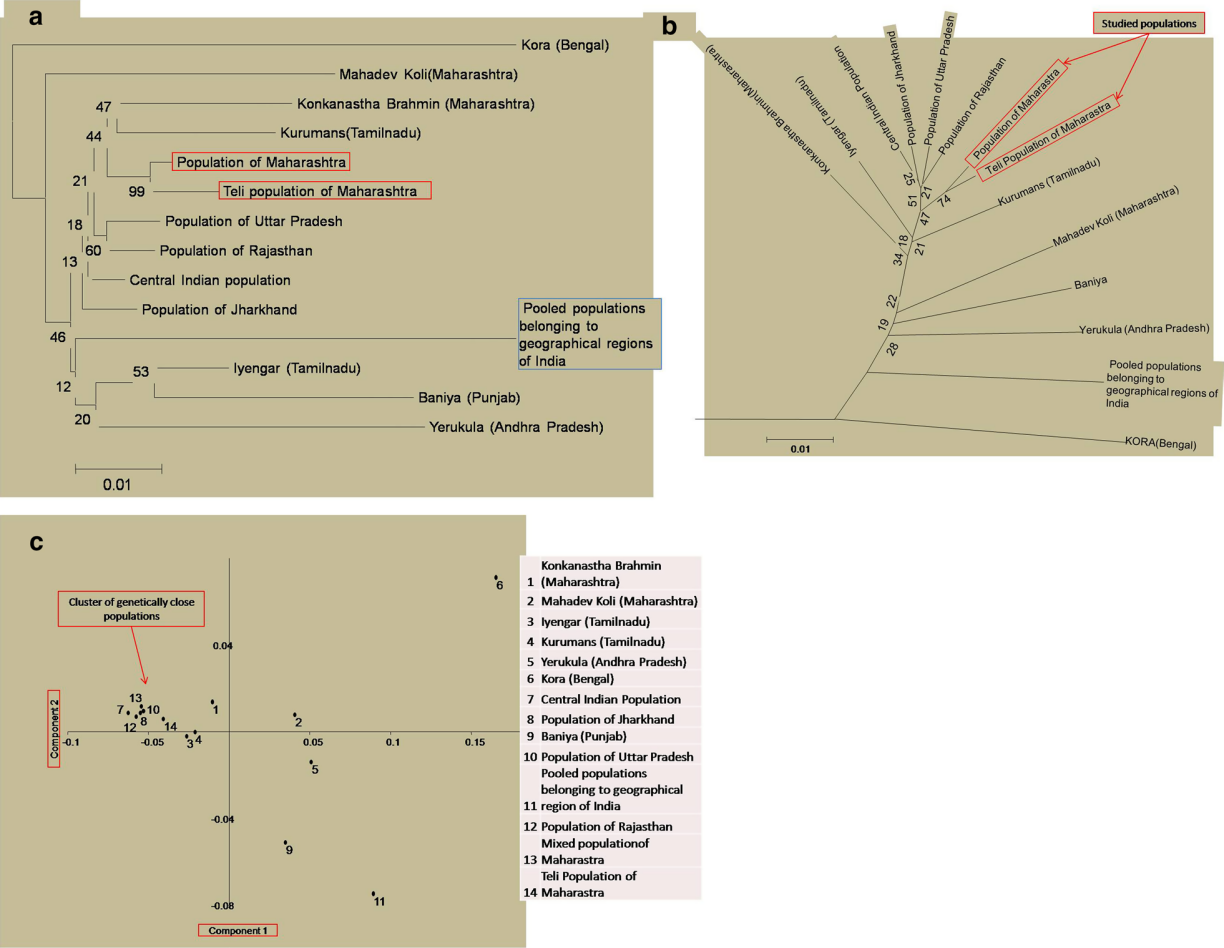
Phylogenetic reconstruction based on **a** Neighbourjoining (NJ) tree with 1000 bootstrap replicates; **b** Maximum likelihood (ML) phylogram with 1000 bootstrap replicates; **c** Principal Component Analysis (PCA) plot based on F*st* genetic distance

A neighbour joining (NJ) tree (Fig. 2a) based on the Nei's genetic distance, constructed using POPTREE-2 software, was used to investigate the genetic affinity between the studied (admixed and*Teli*) populations and the reported Indian populations namely, the *Konkanastha Brahmins* (Maharashtra) [22]; the *Mahadev Kolis* (Maharashtra) [22]; the *Iyengars* (Tamil Nadu) [22]; the *Kurumans* (Tamil Nadu) [22]; the *Yerukulas* (Andhra Pradesh) [23]; the *Koras* (West Bengal) [24]; the *Baniyas* (Punjab) [25]; the population of Jharkhand [26]; the population of Uttar Pradesh [27]; the population of Rajasthan [28]; the populations of Central India [29]; and the pooled populations belonging to the geographical boundaries of India [30]. The NJ tree revealed that the studied admixed and *Teli* populations of Maharashtra pooled into one cluster with the *Konkanastha Brahmin*s of Maharashtra and the *Kurumans* of Tamil Nadu. The populations of Rajasthan, Uttar Pradesh, Madhya Pradesh and Jharkhand also pooled with the studied populations, which might be the result of ancestral relatedness [3, 4]. The *Koras* of West Bengal, the *Baniya*s of Punjab and the pooled populations of Indian geographical region were observed to be the outliers in the NJ tree, which could be attributed to the isolation on the account of distance [31]. The *Mahadev Koli* population of Maharashtra, despite being geographically close to the studied populations, showed genetic distinction, which might be the result of small effective sample size, the founder effect and drift [32]. The maximum likelihood (ML) phylogram (Fig. 2b) showed consistency with the NJ tree with respect to the scattering pattern of the studied and compared populations. In the NJ tree, three nodes out of eleven had the bootstrap values above 50 percent and the three nodes had the bootstrap values of more than 25 percent. In the case of the ML phylogram, out of the eleven nodes, two had the bootstrap values of over 50 percent and four of the nodes had the bootstrap values higher than 25 percent. Similar patterns in the bootstrap values were observed in the NJ tree and the ML phylogram, suggesting a low level of confidence.

In order to validate the genetic relatedness observed in the NJ tree and the ML phylogram with the low bootstrap values, principal component analysis (PCA) and locus-wise F*st* distance calculation between the studied and compared populations were undertaken. In the PCA plot (Fig. 2c), both the studied populations clustered and made patterns similar to those observed in the NJ tree and the ML phylogram. In the case of pair-wise F*st* distance, out of 15 loci, the admixed population of Maharashtra showed significant variations at ten loci with the *Yerukulas* (Andhra Pradesh), at nine loci with the *Koras* (Bengal), at seven loci with the *Mahadev Kolis* (Maharashtra), at four loci with the *Konkanastha Brahmins* (Maharashtra) and the *Baniyas* (Punjab), at three loci with the pooled Indian populations and the population of Rajasthan, at two loci with the *Kurmans* (Tamil Nadu), the Central Indian population and the population of Jharkhand, and at one locus with the *Iyengars* (Tamil Nadu) and the population of Uttar Pradesh. Similarly, the results of pair-wise F*st* distance analyses in the *Teli* population group also showed significant variations at the ten loci with the *Koras* (Bengal), at six loci with the *Mahadev Koli*s (Maharashtra), at four loci with the *Konkanastha Brahmins* (Maharashtra) and the *Yerukulas* (Andhra Pradesh), at two loci with the *Baniyas* (Punjab), the population of Jharkhand, the population of Uttar Pradesh and the pooled Indian populations, and at one locus with the *Iyengars* (Tamil Nadu), the *Kurmans* (Tamil Nadu), the population of Rajasthan and the Central Indian population. No significant variations were observed in the studied populations among all the compared 15 loci (Additional file 2: Table S2). On the contrary, the *Teli* population group showed significant similarities at all compared 15 loci, with the admixed population group (Additional file 3: Table S3). Interestingly, both the studied populations showed a similar pattern of F*st* distances with the compared populations. The mean F*st* value of the studied and compared populations, irrespective of their geographical locations have been shown in Fig. 1a–c.

Overall, the results of the Principal Component Analysis and the F*st* distance study were found to be consistent with each other, and support the genetic relatedness observed in the neighbourjoining tree and the maximum likelihood phylogram.

Since the obtained genetic data showed a high degree of polymorphism and forensic efficacy,it might be useful for forensic DNA application, genetic and genealogical studies, and may enrich the national autosomal STR database.

## Limitations

The small sample size was the main limitation of this study. However, the analyzed samples well explain the polymorphic nature of the studied genetic markers and the genetic affinity of the studied population with the previously reported populations. We further propose the use of larger sample size and Next Generation Sequencing (NGS) studies.

## Supplementary Information

**Additional file 1: Table S1.** Allele frequencies and forensic parameters for the 20 autosomal STR loci in the Teli population of Maharashtra, India (n=69).

**Additional file 2: Table S2.** F*st* pairwise genetic distances between the admixed population of Maharashtra and the compared populations with their corresponding p-valueFst pairwise genetic distances between the admixed population of Maharashtra and the compared populations with their corresponding p-value.

**Additional file 3: Table S3.** F*st* pairwise genetic distances between the* Teli* population of Maharashtra and the compared populations with their corresponding p-value.

## Data Availability

Data sets generated during this study are available from the corresponding author on reasonable request.

## Abbreviations

PCIA: Phenol–chloroform isoamyl alcohol
RT-PCR: Real time—polymerase chain reaction
PIC: Polymorphic information content
PD: Power of discrimination
PE: Power of exclusion
PM: Matching probability
PI: Paternity index
HWE: Hardy–Weinberg equilibrium
Hobs: Observed heterozygosity
Hexp: Expected heterozygosity
NJ: Neighbor joining
PCA: Principle component analysis

## References

[1] Chandramouli C, General R. Census of India 2011. Provisional Popul Total New Delhi Gov India. 2011;

[2] Navaneetham K, Dharmalingam A. Demography and development: preliminary interpretations of the (2011). Census. Econ PolitWkly.

[3] Debortoli G, Abbatangelo C, Ceballos F, Fortes-Lima C, Norton HL, Ozarkar S (2020). Novel insights on demographic history of tribal and caste groups from West Maharashtra (India) using genome-wide data. Sci Rep.

[4] Reich D, Thangaraj K, Patterson N, Price AL, Singh L (2009). Reconstructing Indian population history. Nature.

[5] Jonnalagadda M, Ozarkar S, Ashma R, Kulkarni S (2016). Skin pigmentation variation among populations of West Maharashtra. India Am J Hum Biol.

[6] Malhotra KC. Population structure among the Dhangar caste-cluster of Maharashtra, India. In: The People of South Asia. Springer; 1984. p. 295–324.

[7] Reddy BM, Tripathy V, Kumar V, Alla N (2010). Molecular genetic perspectives on the Indian social structure. Am J Hum Biol.

[8] R. V. Russell. The Tribes and Castes of the Central Provinces of India Volume III. Macmillan And Co., Limited, St. Martins Street London 1916; 2007. 215–224 p. http://www.gutenberg.org/files/22010/22010-h/22010-h.htm

[9] Yilmaz A, Çetin İ. In Silico Prediction of the Effects of Nonsynonymous Single Nucleotide Polymorphisms in the Human Catechol-O-Methyltransferase (COMT) Gene. Cell Biochem Biophys. 2020;1–13. 10.1007/s12013-020-00905-6 32236879

[10] Gopalakrishnan C, Jethi S, Kalsi N, Purohit R (2016). Biophysical aspect of huntingtin protein during polyQ: An in silico insight. Cell BiochemBiophys.

[11] Doss CGP, Rajith B, Rajasekaran R, Srajan J, Nagasundaram N, Debajyoti C (2013). In silico analysis of prion protein mutants: A comparative study by molecular dynamics approach. Cell BiochemBiophys.

[12] Nagasundaram N, Doss CGP (2013). Predicting the impact of single-nucleotide polymorphisms in CDK2–flavopiridol complex by molecular dynamics analysis. Cell BiochemBiophys.

[13] Rickham PP. Human experimentation. Code of ethics of the world medical association. Declaration of Helsinki. Br Med J. 1964;2(5402):177. 10.1136/bmj.2.5402.177 PMC1816102 14150898

[14] Sambrook J, Fritsch EF, Maniatis T. Molecular cloning: a laboratory manual. Cold Spring Harbor, NY: Cold Spring Harbor Laboratory Press; 1989. xxxviii + 1546 pp. https://www.cabdirect.org/cabdirect/abstract/19901616061

[15] Peakall ROD, Smouse PE. GENALEX 6: genetic analysis in Excel. Population genetic software for teaching and research. Mol Ecol Notes. 2006;6(1):288–95. 10.1093/bioinformatics/bts460 PMC3463245 22820204

[16] Tereba A (1999). Tools for analysis of population statistics. Profiles DNA.

[17] Excoffier L, Lischer HEL (2010). Arlequin suite ver 3.5: a new series of programs to perform population genetics analyses under Linux and Windows. MolEcolResour..

[18] Takezaki N, Nei M, Tamura K (2009). POPTREE2: Software for constructing population trees from allele frequency data and computing other population statistics with Windows interface. MolBiolEvol.

[19] Nei M (1972). Genetic distance between populations. Am Nat.

[20] Hammer Ø, Harper DAT, Ryan PD (2001). PAST: paleontological statistics software package for education and data analysis palaeontol. Electronica.

[21] Agrawal S, Khan F (2005). Reconstructing recent human phylogenies with forensic STR loci: a statistical approach. BMC Genet.

[22] Ghosh T, Kalpana D, Mukerjee S, Mukherjee M, Sharma AK, Nath S (2011). Genetic diversity of autosomal STRs in eleven populations of India. Forensic SciInt Genet.

[23] HimaBindu G, Trivedi R, Kashyap VK (2005). Genotypic polymorphisms at fifteen tetranucleotides and two pentanucleotide repeat loci in four tribal populations of Andhra Pradesh, southern India. J Forensic Sci.

[24] Singh A, Trivedi R, Kashyap VK (2006). Genetic polymorphism at 15 tetrameric short tandem repeat loci in four aboriginal tribal populations of Bengal. J Forensic Sci.

[25] Giroti R, Talwar I (2013). Diversity and differentiation in Khatris, Banias and Jat Sikhs of Punjab: a study with forensic microsatellites. Ind J Phys Anthr Hum Genet.

[26] Imam J, Reyaz R, Singh RS, Bapuly AK, Shrivastava P (2018). Genomic portrait of population of Jharkhand, India, drawn with 15 autosomal STRs and 17 Y-STRs. Int J Legal Med.

[27] Shrivastava P, Kaitholia K, Kumawat RK, Dixit S, Dash HR, Srivastava A (2019). Forensic effectiveness and genetic distribution of 23 autosomal STRs included in Verifiler Plus TM multiplex in a population sample from Madhya Pradesh. India. Int J Legal Med..

[28] Kumawat RK, Shrivastava P, Shrivastava D, Mathur GK, Dixit S (2020). Genomic blueprint of population of Rajasthan based on autosomal STR markers. Ann Hum Biol..

[29] Shrivastava P, Jain T, Trivedi VB (2015). Genetic polymorphism study at 15 autosomal locus in central Indian population. Springerplus..

[30] Singh M, Nandineni MR (2017). Population genetic analyses and evaluation of 22 autosomal STRs in Indian populations. Int J Legal Med.

[31] Chaubey G, Metspalu M, Kivisild T, Villems R (2007). Peopling of South Asia: Investigating the caste-tribe continuum in India. BioEssays.

[32] Palo JU, Ulmanen I, Lukka M, Ellonen P, Sajantila A (2009). Genetic markers and population history: Finland revisited. Eur J Hum Genet.

