# Supplementary material for: A study of genomic diversity in populations of Maharashtra, India, inferred from 20 autosomal STR markers
Source: BMC Res Notes. 2021 Feb 23;14:69. doi: 10.1186/s13104-021-05485-z (PMC7903603; doi:10.1186/s13104-021-05485-z)
Supplement: Supplementary file 1 — Additional file 1: Table S1. Allele frequencies and forensic parameters for the 20 autosomal STR loci in the Teli population of Maharashtra, India (n=69). [file 13104_2021_5485_MOESM1_ESM.docx]

| Table S1: Allele frequencies and forensic parameters for the 20 autosomal STR loci in the *Teli* population of Maharashtra, India (n=69) | | | | | | | | | | | | | | | | | | | | |
| --- | --- | --- | --- | --- | --- | --- | --- | --- | --- | --- | --- | --- | --- | --- | --- | --- | --- | --- | --- | --- |
| **Allele** | **D3S1358** | **D1S1656** | **D6S1043** | **D13S317** | **Penta E** | **D16S539** | **D18S51** | **D2S1338** | **CSF1PO** | **Penta D** | **TH01** | **vWA** | **D21S11** | **D7S820** | **D5S818** | **TPOX** | **D8S1179** | **D12S391** | **D19S433** | **FGA** |
| **5** | - | - | - | - | 0.043 | - | - | - | - | - | - | - | - | - | - | - | - | - | - | - |
| **6** | - | - | - | - | - | - | - | - | - | - | 0.312 | - | - | - | - | - | - | - | - | - |
| **7** | - | - | - | 0.022 | 0.072 | - | - | - | - | 0.022 | 0.109 | - | - | 0.022 | - | - | - | - | - | - |
| **8** | - | 0.022 | - | 0.138 | - | 0.022 | - | - | - | 0.007 | 0.174 | - | - | 0.239 | - | 0.275 | 0.007 | - | - | - |
| **9** | - | - | 0.007 | 0.159 | 0.014 | 0.232 | - | - | 0.007 | 0.167 | 0.232 | - | - | 0.072 | 0.022 | 0.174 | - | - | - | - |
| **9.3** | - | - | - | - | - | - | - | - | - | - | 0.167 | - | - | - | - | - | - | - | - | - |
| **10** | - | 0.007 | - | 0.101 | 0.043 | 0.116 | 0.007 | - | 0.217 | 0.297 | 0.007 | - | - | 0.203 | 0.159 | 0.101 | 0.196 | - | - | - |
| **11** | - | 0.116 | 0.268 | 0.283 | 0.174 | 0.275 | 0.036 | - | 0.333 | 0.246 | - | - | - | 0.283 | 0.290 | 0.435 | 0.145 | - | 0.007 | - |
| **12** | - | 0.080 | 0.246 | 0.225 | 0.087 | 0.181 | 0.116 | - | 0.319 | 0.138 | - | - | - | 0.138 | 0.377 | 0.014 | 0.036 | - | 0.101 | - |
| **13** | - | 0.130 | 0.094 | 0.051 | 0.087 | 0.159 | 0.152 | - | 0.109 | 0.080 | - | - | - | 0.022 | 0.145 | - | 0.174 | - | 0.326 | - |
| **13.2** | - | - | - | - | - | - | - | - | - | - | - | - | - | - | - | - | - | - | 0.014 | - |
| **14** | 0.072 | 0.130 | 0.080 | 0.022 | 0.065 | 0.014 | 0.283 | - | 0.007 | 0.036 | - | 0.094 | - | 0.022 | 0.007 | - | 0.181 | - | 0.217 | - |
| **14.2** | - | - | - | - | - | - | - | - | - | - | - | - | - | - | - | - | - | - | 0.101 | - |
| **15** | 0.304 | 0.167 | 0.007 | - | 0.043 | - | 0.167 | - | 0.007 | 0.007 | - | 0.174 | - | - | - | - | 0.196 | - | 0.101 | - |
| **15.2** | - | - | - | - | - | - | - | - | - | - | - | - | - | - | - | - | - | - | 0.036 | - |
| **15.3** | - | 0.022 | - | - | - | - | - | - | - | - | - | - | - | - | - | - | - | - | - | - |
| **16** | 0.239 | 0.130 | - | - | 0.101 | - | 0.072 | - | - | - | - | 0.297 | - | - | - | - | 0.058 | 0.014 | 0.058 | - |
| **16.2** | - | - | - | - | - | - | - | - | - | - | - | - | - | - | - | - | - | - | 0.036 | - |
| **16.3** | - | 0.051 | - | - | - | - | - | - | - | - | - | - | - | - | - | - | - | - | - | - |
| **16.4** | - | - | - | - | 0.007 | - | - | - | - | - | - | - | - | - | - | - | - | - | - | - |
| **17** | 0.239 | 0.072 | 0.051 | - | 0.080 | - | 0.065 | 0.007 | - | - | - | 0.232 | - | - | - | - | 0.007 | 0.130 | - | - |
| **17.3** | - | 0.036 | - | - | - | - | - | - | - | - | - | - | - | - | - | - | - | - | - | - |
| **18** | 0.145 | 0.014 | 0.109 | - | 0.109 | - | 0.036 | 0.138 | - | - | - | 0.174 | - | - | - | - | - | 0.239 | - | - |
| **18.3** | - | 0.007 | - | - | - | - | - | - | - | - | - | - | - | - | - | - | - | 0.022 | - | - |
| **19** | - | - | 0.087 | - | 0.014 | - | 0.022 | 0.181 | - | - | - | 0.029 | - | - | - | - | - | 0.130 | - | 0.065 |
| **19.2** | - | - | - | - | - | - | - | - | - | - | - | - | - | - | - | - | - | 0.007 | - | 0.007 |
| **19.3** | - | 0.014 | - | - | - | - | - | - | - | - | - | - | - | - | - | - | - | 0.007 | - | - |
| **20** | - | - | 0.051 | - | 0.043 | - | 0.029 | 0.159 | - | - | - | - | - | - | - | - | - | 0.130 | - | 0.101 |
| **21** | - | - | - | - | 0.007 | - | 0.014 | 0.036 | - | - | - | - | - | - | - | - | - | 0.101 | - | 0.159 |
| **22** | - | - | - | - | - | - | - | 0.072 | - | - | - | - | - | - | - | - | - | 0.101 | - | 0.145 |
| **22.2** | - | - | - | - | - | - | - | - | - | - | - | - | - | - | - | - | - | - | - | 0.007 |
| **23** | - | - | - | - | 0.007 | - | - | 0.203 | - | - | - | - | - | - | - | - | - | 0.036 | - | 0.159 |
| **24** | - | - | - | - | - | - | - | 0.123 | - | - | - | - | - | - | - | - | - | 0.036 | - | 0.123 |
| **24.2** | - | - | - | - | - | - | - | - | - | - | - | - | - | - | - | - | - | - | - | 0.007 |
| **25** | - | - | - | - | - | - | - | 0.058 | - | - | - | - | - | - | - | - | - | 0.014 | - | 0.167 |
| **25.2** | - | - | - | - | - | - | - | - | - | - | - | - | - | - | - | - | - | - | - | 0.007 |
| **26** | - | - | - | - | - | - | - | 0.014 | - | - | - | - | - | - | - | - | - | 0.029 | - | 0.043 |
| **27** | - | - | - | - | - | - | - | - | - | - | - | - | 0.014 | - | - | - | - | - | - | 0.007 |
| **28** | - | - | - | - | - | - | - | - | - | - | - | - | 0.145 | - | - | - | - | - | - | - |
| **28.3** | - | - | - | - | - | - | - | - | - | - | - | - | 0.007 | - | - | - | - | - | - | - |
| **29** | - | - | - | - | - | - | - | 0.007 | - | - | - | - | 0.181 | - | - | - | - | - | - | - |
| **29.2** | - | - | - | - | - | - | - | - | - | - | - | - | 0.007 | - | - | - | - | - | - | - |
| **30** | - | - | - | - | - | - | - | - | - | - | - | - | 0.210 | - | - | - | - | - | - | - |
| **30.2** | - | - | - | - | - | - | - | - | - | - | - | - | 0.014 | - | - | - | - | - | - | - |
| **31** | - | - | - | - | - | - | - | - | - | - | - | - | 0.051 | - | - | - | - | - | - | - |
| **31.1** | - | - | - | - | - | - | - | - | - | - | - | - | 0.007 | - | - | - | - | - | - | - |
| **31.2** | - | - | - | - | - | - | - | - | - | - | - | - | 0.072 | - | - | - | - | - | - | - |
| **31.3** | - | - | - | - | - | - | - | - | - | - | - | - | 0.007 | - | - | - | - | - | - | - |
| **32** | - | - | - | - | - | - | - | - | - | - | - | - | 0.007 | - | - | - | - | - | - | - |
| **32.2** | - | - | - | - | - | - | - | - | - | - | - | - | 0.174 | - | - | - | - | - | - | - |
| **32.3** | - | - | - | - | - | - | - | - | - | - | - | - | 0.007 | - | - | - | - | - | - | - |
| **33.2** | - | - | - | - | - | - | - | - | - | - | - | - | 0.072 | - | - | - | - | - | - | - |
| **34.2** | - | - | - | - | - | - | - | - | - | - | - | - | 0.022 | - | - | - | - | - | - | - |
| Pm | 0.090 | 0.040 | 0.061 | 0.072 | 0.026 | 0.072 | 0.064 | 0.051 | 0.122 | 0.078 | 0.098 | 0.085 | 0.048 | 0.098 | 0.138 | 0.151 | 0.062 | 0.038 | 0.071 | 0.041 |
| PIC | 0.728 | 0.881 | 0.807 | 0.786 | 0.902 | 0.768 | 0.825 | 0.840 | 0.679 | 0.767 | 0.745 | 0.755 | 0.842 | 0.766 | 0.681 | 0.644 | 0.813 | 0.853 | 0.787 | 0.854 |
| Hexp | 0.767 | 0.891 | 0.828 | 0.811 | 0.909 | 0.798 | 0.842 | 0.856 | 0.728 | 0.796 | 0.779 | 0.788 | 0.857 | 0.796 | 0.727 | 0.694 | 0.835 | 0.867 | 0.809 | 0.869 |
| Hobs | 0.725 | 0.942 | 0.797 | 0.812 | 0.884 | 0.768 | 0.855 | 0.899 | 0.739 | 0.797 | 0.855 | 0.797 | 0.884 | 0.884 | 0.696 | 0.725 | 0.841 | 0.855 | 0.797 | 0.841 |
| P-value | 0.726 | 0.139 | 0.392 | 0.372 | 0.476 | 0.901 | 0.032 | 0.422 | 0.897 | 0.723 | 0.320 | 0.818 | 0.524 | 0.066 | 0.004 | 0.245 | 0.235 | 0.934 | 0.212 | 0.452 |
| PI | 2.464 | 3.450 | 2.464 | 2.464 | 3.136 | 2.654 | 4.313 | 1.917 | 1.816 | 1.816 | 3.450 | 3.136 | 4.929 | 4.313 | 3.450 | 2.464 | 4.313 | 8.625 | 2.156 | 1.643 |
| PE | 0.467 | 0.882 | 0.594 | 0.621 | 0.763 | 0.541 | 0.705 | 0.792 | 0.491 | 0.594 | 0.705 | 0.594 | 0.763 | 0.763 | 0.422 | 0.467 | 0.676 | 0.705 | 0.594 | 0.676 |
| PD | 0.910 | 0.960 | 0.939 | 0.928 | 0.974 | 0.928 | 0.936 | 0.949 | 0.878 | 0.922 | 0.902 | 0.915 | 0.952 | 0.902 | 0.862 | 0.849 | 0.938 | 0.962 | 0.929 | 0.959 |
| Pm- Matching Probability, PIC- Polymorphic information content, Hexp- Expected Heterozygosity,Hobs- Observed heterozygosity, P value- HWE test, PI- Paternity index, PE- Power of exclusion, PD- Power of discrimination | | | | | | | | | | | | | | | | | | | | |
