# Supplementary material for: A study of genomic diversity in populations of Maharashtra, India, inferred from 20 autosomal STR markers
Source: BMC Res Notes. 2021 Feb 23;14:69. doi: 10.1186/s13104-021-05485-z (PMC7903603; doi:10.1186/s13104-021-05485-z)
Supplement: Supplementary file 2 — Additional file 2: Table S2. Fst pairwise genetic distances between the admixed population of Maharashtra and the compared populations with their corresponding p-valueFst pairwise genetic distances between the admixed population of Maharashtra and the compared populations with their corresponding p-value. [file 13104_2021_5485_MOESM2_ESM.docx]

| Table S2: F*st* pairwise genetic distances between the admixed population of Maharashtra and the compared populations with their corresponding p-value | | | | | | | | | | | | |
| --- | --- | --- | --- | --- | --- | --- | --- | --- | --- | --- | --- | --- |
| **Admixed Population of Maharashtra v/s** | **Konkanastha Brahmin** | | **MahadevKoli** | | **Iyengar** | | | **Kurumans** | | | **Yerukula** | |
|  | **(Maharashtra)** | | **(Maharashtra)** | | **(Tamilnadu)** | | | **(Tamilnadu)** | | | **(Andhra Pradesh)** | |
|  | **Fst** | **p-value** | **Fst** | **p-value** | **Fst** | **p-value** | | **Fst** | | **p-value** | **Fst** | **p-value** |
| **D8S1179** | 0.0077 | 0.02703+-0.0194 | 0.0018 | 0.26126+-0.0438 | 0.0074 | 0.03604+-0.0201 | | -0.0468 | | 0.95495+-0.0203 | 0.0240 | **0.00000+-0.0000** |
| **D21S11** | 0.0011 | 0.24324+-0.0451 | 0.0003 | 0.34234+-0.0379 | 0.0459 | **0.00000+-0.0000** | | 0.0054 | | 0.03604+-0.0148 | 0.0142 | **0.00000+-0.0000** |
| **D7S820** | 0.0092 | 0.01802+-0.0182 | 0.0011 | 0.32432+-0.0338 | -0.0024 | 0.72973+-0.0371 | | 0.0023 | | 0.18919+-0.0286 | 0.0115 | **0.00000+-0.0000** |
| **CSF1PO** | 0.0190 | 0.00901+-0.0091 | 0.0002 | 0.36937+-0.0438 | -0.0015 | 0.46847+-0.0379 | | 0.0039 | | 0.09009+-0.0235 | -0.0005 | 0.35135+-0.0613 |
| **D19S433** | 0.0047 | 0.04505+-0.0203 | 0.0182 | **0.00000+-0.0000** | 0.0013 | 0.22523+-0.0339 | | -0.0007 | | 0.51351+-0.0360 | 0.0152 | **0.00000+-0.0000** |
| **vWA** | 0.0042 | 0.12613+-0.0278 | 0.0226 | **0.00000+-0.0000** | 0.0019 | 0.20721+-0.0408 | | 0.0159 | | **0.00000+-0.0000** | 0.0286 | **0.00000+-0.0000** |
| **TPOX** | 0.0010 | 0.31532+-0.0412 | 0.0150 | 0.01802+-0.0121 | 0.0075 | 0.07207+-0.0378 | | 0.0098 | | 0.05405+-0.0201 | -0.0022 | 0.64865+-0.0354 |
| **D18S51** | 0.0174 | **0.00000+-0.0000** | 0.0184 | **0.00000+-0.0000** | -0.0035 | 0.93694+-0.0365 | | 0.0056 | | 0.04505+-0.0152 | 0.0077 | 0.00901+-0.0091 |
| **D3S1358** | -0.0025 | 0.70270+-0.0403 | 0.0148 | **0.00000+-0.0000** | 0.0084 | 0.05405+-0.0201 | | 0.0000 | | 0.38739+-0.0490 | 0.0325 | **0.00000+-0.0000** |
| **THO1** | 0.0046 | 0.10811+-0.0264 | 0.0049 | 0.09009+-0.0192 | -0.0009 | 0.53153+-0.0417 | | 0.0001 | | 0.35135+-0.0370 | 0.0154 | **0.00000+-0.0000** |
| **D13S317** | 0.0024 | 0.12613+-0.0388 | 0.0348 | **0.00000+-0.0000** | 0.0085 | 0.01802+-0.0121 | | 0.0021 | | 0.23423+-0.0609 | 0.0148 | **0.00000+-0.0000** |
| **D16S539** | 0.0099 | 0.01802+-0.0121 | 0.0157 | **0.00000+-0.0000** | -0.0029 | 0.78378+-0.0334 | | 0.0191 | | **0.00000+-0.0000** | 0.0039 | 0.09009+-0.0235 |
| **D2S1338** | 0.0096 | **0.00000+-0.0000** | 0.0151 | **0.00000+-0.0000** | 0.0006 | 0.31532+-0.0654 | | -0.0023 | | 0.72072+-0.0525 | 0.0296 | **0.00000+-0.0000** |
| **D5S818** | 0.1595 | **0.00000+-0.0000** | 0.0054 | 0.15315+-0.0237 | -0.0027 | 0.72072+-0.0359 | | 0.0087 | | 0.04505+-0.0244 | 0.0166 | **0.00000+-0.0000** |
| **FGA** | 0.0181 | **0.00000+-0.0000** | 0.0045 | 0.06306+-0.0305 | -0.0002 | 0.47748+-0.0360 | | 0.0051 | | 0.10811+-0.0378 | 0.0109 | 0.00901+-0.0091 |
| Bold values with significant p values (p value >0.0033) of the studied population with other populations. | | | | | | | | | | | | |
|  |  |  |  |  |  |  | |  | |  |  |  |
| **Table S2: Fst pairwise genetic distances between mixed population of Maharashtra and compared populations with their corresponding p-value** | | | | | | | | | | | | |
| **Mixed Population of Maharashtra v/s** | **Kora** | | **Central Indian Population** | | **Population Of Jharkhand** | | | **Baniya** | | | **Population of Uttar Pradesh** | |
|  | **(Bengal)** | | **(Madhya Pradesh)** | | **(Jharkhand)** | | | **(Punjab)** | | | **(Uttar Pradesh)** | |
|  | **Fst** | **p-value** | **Fst** | **p-value** | **Fst** | **p-value** | | **Fst** | | **p-value** | **Fst** | **p-value** |
| **D8S1179** | 0.01718 | **0.00000+-0.0000** | 0.00167 | 0.08108+-0.0252 | 0.00352 | 0.05405+-0.0242 | | 0.01005 | | 0.00901+-0.0091 | 0.00215 | 0.16216+-0.0445 |
| **D21S11** | 0.0238 | **0.00000+-0.0000** | 0.00207 | 0.00901+-0.0091 | 0.00497 | 0.01802+-0.0182 | | 0.14401 | | **0.00000+-0.0000** | 0.00358 | 0.01802+-0.0121 |
| **D7S820** | 0.00012 | 0.39640+-0.0433 | -0.00073 | 0.68468+-0.0364 | -0.00111 | 0.69369+-0.0430 | | -0.00315 | | 0.79279+-0.0466 | -0.00114 | 0.63964+-0.0252 |
| **CSF1PO** | 0.02767 | **0.00000+-0.0000** | -0.00022 | 0.46847+-0.0354 | 0.00057 | 0.21622+-0.0454 | | -0.00187 | | 0.54955+-0.0478 | 0.00154 | 0.23423+-0.0454 |
| **D19S433** | 0.00801 | 0.00901+-0.0091 | 0.00709 | **0.00000+-0.0000** | 0.00347 | 0.01802+-0.0121 | | -0.00182 | | 0.57658+-0.0364 | 0.0057 | 0.00901+-0.0091 |
| **vWA** | 0.10699 | **0.00000+-0.0000** | 0.00166 | 0.09910+-0.0286 | 0.00332 | 0.05405+-0.0278 | | 0.01259 | | **0.00000+-0.0000** | 0.00585 | 0.02703+-0.0139 |
| **TPOX** | 0.02377 | **0.00000+-0.0000** | 0.00289 | 0.07207+-0.0227 | -0.00154 | 0.73874+-0.0379 | | 0.00412 | | 0.19820+-0.0379 | 0.0126 | **0.00000+-0.0000** |
| **D18S51** | 0.02603 | **0.00000+-0.0000** | -0.00013 | 0.38739+-0.0490 | -0.00107 | 0.77477+-0.0579 | | 0.01725 | | **0.00000+-0.0000** | -0.00105 | 0.72973+-0.0598 |
| **D3S1358** | 0.00389 | 0.05405+-0.0242 | 0.00001 | 0.33333+-0.0430 | 0.00023 | 0.36937+-0.0515 | | 0.24439 | | **0.00000+-0.0000** | 0.00449 | 0.04505+-0.0152 |
| **THO1** | 0.05894 | **0.00000+-0.0000** | -0.00009 | 0.43243+-0.0504 | 0.00482 | 0.02703+-0.0194 | | 0.00666 | | 0.13514+-0.0311 | -0.00064 | 0.56757+-0.0543 |
| **D13S317** | 0.02984 | **0.00000+-0.0000** | 0.00048 | 0.25225+-0.0379 | 0.00348 | 0.06306+-0.0273 | | 0.01248 | | 0.00901+-0.0091 | 0.00103 | 0.30631+-0.0639 |
| **D16S539** | 0.00755 | 0.00901+-0.0091 | 0.00122 | 0.14414+-0.0433 | 0.03227 | **0.00000+-0.0000** | | 0.0052 | | 0.09910+-0.0286 | 0.00252 | 0.14414+-0.0309 |
| **D2S1338** | 0.02759 | **0.00000+-0.0000** | 0.00076 | 0.18919+-0.0286 | 0.03214 | **0.00000+-0.0000** | | 0.00289 | | 0.18919+-0.0394 | 0.00081 | 0.22523+-0.0389 |
| **D5S818** | 0.00302 | 0.09910+-0.0212 | 0.02035 | **0.00000+-0.0000** | 0.0029 | 0.11712+-0.0237 | | 0.00493 | | 0.09910+-0.0163 | 0.00128 | 0.22523+-0.0434 |
| **FGA** | 0.00454 | 0.01802+-0.0121 | 0.00097 | 0.14414+-0.0309 | 0.00352 | 0.02703+-0.0194 | | 0.00671 | | 0.02703+-0.0139 | 0.00234 | 0.09009+-0.0303 |
| Bold values with significant p values (p value >0.0033) of the studied population with other populations. | | | | | | | | | | | | |
|  |  |  |  |  |  |  | | |  |  |  |  |
| **Table S2: Fst pairwise genetic distances between mixed population of Maharashtra and compared populations with their corresponding p-value** | | | | | | | | |  |  |  |  |
| **Mixed Population of Maharashtra v/s** | **Pooled populations belonging to geographical region of India** | | **Population of Rajasthan** | | **Teli population** | | | |  |  |  |  |
|  | **(India)** | | **(Rajasthan)** | | **(Maharashtra)** | | | |  |  |  |  |
|  | **Fst** | **p-value** | **Fst** | **p-value** | **Fst** | | **p-value** | |  |  |  |  |
| **D8S1179** | 0.0367 | **0.00000+-0.0000** | 0.00568 | **0.00000+-0.0000** | -0.0029 | | 0.77477+-0.0310 | |  |  |  |  |
| **D21S11** | 0.0022 | 0.03604+-0.0201 | 0.00007 | 0.42342+-0.0692 | -0.0041 | | 0.98198+-0.0096 | |  |  |  |  |
| **D7S820** | -0.0007 | 0.64865+-0.0402 | 0.00226 | 0.07207+-0.0227 | -0.0042 | | 0.98198+-0.0096 | |  |  |  |  |
| **CSF1PO** | -0.0015 | 0.81982+-0.0253 | 0.0033 | 0.06306+-0.0194 | -0.0029 | | 0.66667+-0.0389 | |  |  |  |  |
| **D19S433** | 0.0065 | **0.00000+-0.0000** | 0.00095 | 0.20721+-0.0360 | -0.0022 | | 0.76577+-0.0340 | |  |  |  |  |
| **vWA** | -0.0002 | 0.45045+-0.0235 | 0.00446 | **0.00000+-0.0000** | 0.0046 | | 0.06306+-0.0194 | |  |  |  |  |
| **TPOX** | 0.0006 | 0.27928+-0.0613 | 0.0037 | 0.06306+-0.0273 | -0.0046 | | 0.94595+-0.0246 | |  |  |  |  |
| **D18S51** | 0.1090 | **0.00000+-0.0000** | 0.00212 | 0.07207+-0.0264 | -0.0028 | | 0.84685+-0.0244 | |  |  |  |  |
| **D3S1358** | 0.0013 | 0.16216+-0.0353 | 0.00137 | 0.21622+-0.0411 | -0.0023 | | 0.62162+-0.0345 | |  |  |  |  |
| **THO1** | 0.0000 | 0.36036+-0.0332 | -0.00014 | 0.43243+-0.0485 | 0.0002 | | 0.37838+-0.0354 | |  |  |  |  |
| **D13S317** | 0.0007 | 0.28829+-0.0445 | -0.00029 | 0.45045+-0.0489 | -0.0005 | | 0.42342+-0.0408 | |  |  |  |  |
| **D16S539** | 0.0010 | 0.18018+-0.0469 | 0.00402 | **0.00000+-0.0000** | 0.0004 | | 0.34234+-0.0354 | |  |  |  |  |
| **D2S1338** | 0.0002 | 0.39640+-0.0694 | -0.00054 | 0.63063+-0.0407 | -0.0027 | | 0.80180+-0.0345 | |  |  |  |  |
| **D5S818** | 0.0050 | 0.01802+-0.0121 | 0.0033 | 0.05405+-0.0201 | -0.0033 | | 0.81081+-0.0359 | |  |  |  |  |
| **FGA** | 0.0001 | 0.36036+-0.0525 | 0.0017 | 0.14414+-0.0201 | -0.0035 | | 0.97297+-0.0125 | |  |  |  |  |
| Bold values with significant p values (p value >0.0033) of the studied population with other populations. | | | | | | | | |  |  |  |  |
