# Supplementary material for: A study of genomic diversity in populations of Maharashtra, India, inferred from 20 autosomal STR markers
Source: BMC Res Notes. 2021 Feb 23;14:69. doi: 10.1186/s13104-021-05485-z (PMC7903603; doi:10.1186/s13104-021-05485-z)
Supplement: Supplementary file 3 — Additional file 3: Table S3. Fst pairwise genetic distances between the Teli population of Maharashtra and the compared populations with their corresponding p-value. [file 13104_2021_5485_MOESM3_ESM.docx]

| Table S3: F*st* pairwise genetic distances between the *Teli*  population of Maharashtra and the compared populations with their corresponding p-value | | | | | | | | | | | | | | | | | |
| --- | --- | --- | --- | --- | --- | --- | --- | --- | --- | --- | --- | --- | --- | --- | --- | --- | --- |
| **Teli Population (Maharashtra) v/s** | **Konkanastha Brahmin** | | | | **MahadevKoli** | | | **Iyengar** | | | **Kurumans** | | | | | **Yerukula** | |
|  | **(Maharashtra)** | | | | **(Maharashtra)** | | | **(Tamilnadu)** | | | **(Tamilnadu)** | | | | | **(Andhra Pradesh)** | |
|  | **Fst** | | **p-value** | | **Fst** | **p-value** | | **Fst** | | **p-value** | **Fst** | | | **p-value** | | **Fst** | **p-value** |
| **D8S1179** | 0.01693 | | 0.00901+-0.0091 | | -0.00085 | 0.39640+-0.0594 | | 0.0052 | | 0.08108+-0.0252 | -0.04302 | | | 0.89189+-0.0345 | | 0.02893 | **0.00000+-0.0000** |
| **D21S11** | -0.00033 | | 0.49550+-0.0390 | | -0.00166 | 0.57658+-0.0493 | | 0.04236 | | **0.00000+-0.0000** | 0.0017 | | | 0.27928+-0.0478 | | 0.01339 | 0.00901+-0.0091 |
| **D7S820** | 0.00815 | | 0.06306+-0.0194 | | 0.00043 | 0.38739+-0.0678 | | -0.00465 | | 0.85586+-0.0246 | -0.00296 | | | 0.67568+-0.0455 | | 0.01149 | 0.00901+-0.0091 |
| **CSF1PO** | 0.02981 | | **0.00000+-0.0000** | | 0.00239 | 0.27027+-0.0332 | | -0.00394 | | 0.73874+-0.0379 | 0.00657 | | | 0.17117+-0.0438 | | 0.00533 | 0.08108+-0.0212 |
| **D19S433** | 0.00179 | | 0.31532+-0.0529 | | 0.01558 | **0.00000+-0.0000** | | 0.00207 | | 0.14414+-0.0411 | 0.00073 | | | 0.24324+-0.0333 | | 0.01297 | 0.02703+-0.0139 |
| **vWA** | 0.02228 | | 0.00901+-0.0091 | | 0.02451 | **0.00000+-0.0000** | | 0.01768 | | 0.01802+-0.0121 | 0.03624 | | | **0.00000+-0.0000** | | 0.04657 | **0.00000+-0.0000** |
| **TPOX** | 0.00218 | | 0.29730+-0.0360 | | 0.0133 | 0.05405+-0.0278 | | 0.009 | | 0.09009+-0.0235 | 0.01149 | | | 0.02703+-0.0139 | | -0.00413 | 0.81081+-0.0359 |
| **D18S51** | 0.01854 | | **0.00000+-0.0000** | | 0.0197 | **0.00000+-0.0000** | | -0.00495 | | 0.90991+-0.0287 | 0.01044 | | | 0.02703+-0.0139 | | 0.00682 | 0.09009+-0.0359 |
| **D3S1358** | 0.0019 | | 0.26126+-0.0344 | | 0.01777 | 0.00901+-0.0091 | | 0.00744 | | 0.08108+-0.0316 | 0.00774 | | | 0.10811+-0.0297 | | 0.01756 | 0.00901+-0.0091 |
| **THO1** | 0.00058 | | 0.34234+-0.0424 | | 0.02095 | **0.00000+-0.0000** | | 0.0047 | | 0.09910+-0.0370 | -0.00053 | | | 0.41441+-0.0433 | | 0.00754 | 0.09009+-0.0303 |
| **D13S317** | 0.00138 | | 0.25225+-0.0466 | | 0.05778 | **0.00000+-0.0000** | | 0.00787 | | 0.09910+-0.0286 | 0.00331 | | | 0.14414+-0.0278 | | 0.01856 | 0.00901+-0.0091 |
| **D16S539** | 0.01834 | | 0.01802+-0.0121 | | 0.03966 | **0.00000+-0.0000** | | -0.00316 | | 0.73874+-0.0327 | 0.01042 | | | 0.06306+-0.0305 | | 0.00958 | 0.01802+-0.0121 |
| **D2S1338** | 0.0094 | | 0.02703+-0.0139 | | 0.00884 | 0.02703+-0.0139 | | 0.00016 | | 0.39640+-0.0454 | 0.00045 | | | 0.27928+-0.0394 | | 0.02732 | **0.00000+-0.0000** |
| **D5S818** | 0.16634 | | **0.00000+-0.0000** | | 0.00598 | 0.09009+-0.0303 | | -0.00012 | | 0.40541+-0.0389 | 0.01757 | | | 0.03604+-0.0148 | | 0.02374 | **0.00000+-0.0000** |
| **FGA** | 0.01968 | | **0.00000+-0.0000** | | 0.00022 | 0.34234+-0.0485 | | 0.00045 | | 0.33333+-0.0385 | 0.00569 | | | 0.10811+-0.0264 | | 0.01155 | 0.01802+-0.0121 |
| Bold values with significant p values (p value >0.003) of the studied population with other populations. | | | | | | | | | | | | | | | | | |
|  |  | |  | |  |  | |  | |  |  | | |  | |  |  |
| **Table S3: Fst pairwise genetic distances between Teli population of Maharashtra and compared populations with their corresponding p-value** | | | | | | | | | | | | | | | | | |
| **Teli Population (Maharashtra) v/s** | **Kora** | | | | **Central Indian Population** | | | **Population Of Jharkhand** | | | **Baniya** | | | | | **Population of Uttar Pradesh** | |
|  | **(Bengal)** | | | | **(Madhya Pradesh)** | | | **(Jharkhand)** | | | **(Punjab)** | | | | | **(Uttar Pradesh)** | |
|  | **Fst** | | **p-value** | | **Fst** | **p-value** | | **Fst** | | **p-value** | **Fst** | | | **p-value** | | **Fst** | **p-value** |
| **D8S1179** | 0.01756 | | **0.00000+-0.0000** | | 0.00353 | 0.09910+-0.0212 | | 0.00638 | | 0.02703+-0.0139 | 0.00886 | | | 0.07207+-0.0326 | | 0.00297 | 0.09910+-0.0212 |
| **D21S11** | 0.02347 | | **0.00000+-0.0000** | | -0.00093 | 0.61261+-0.0539 | | 0.00262 | | 0.14414+-0.0364 | 0.14305 | | | **0.00000+-0.0000** | | 0.00067 | 0.33333+-0.0490 |
| **D7S820** | -0.00125 | | 0.49550+-0.0512 | | -0.00175 | 0.76577+-0.0390 | | -0.00332 | | 0.89189+-0.0318 | -0.00377 | | | 0.69369+-0.0334 | | -0.00083 | 0.48649+-0.0667 |
| **CSF1PO** | 0.02597 | | **0.00000+-0.0000** | | 0.00109 | 0.19820+-0.0539 | | 0.00606 | | 0.07207+-0.0227 | -0.00007 | | | 0.38739+-0.0334 | | 0.00565 | 0.14414+-0.0309 |
| **D19S433** | 0.01179 | | 0.02703+-0.0139 | | 0.00056 | 0.32432+-0.0578 | | 0.00076 | | 0.25225+-0.0326 | -0.0043 | | | 0.74775+-0.0305 | | 0.0047 | 0.08108+-0.0212 |
| **vWA** | 0.13322 | | **0.00000+-0.0000** | | 0.00661 | 0.01802+-0.0121 | | 0.0087 | | 0.02703+-0.0194 | 0.00191 | | | 0.29730+-0.0408 | | 0.01297 | **0.00000+-0.0000** |
| **TPOX** | 0.01907 | | **0.00000+-0.0000** | | 0.00336 | 0.15315+-0.0273 | | -0.00338 | | 0.79279+-0.0327 | 0.0063 | | | 0.14414+-0.0411 | | 0.01479 | **0.00000+-0.0000** |
| **D18S51** | 0.03282 | | **0.00000+-0.0000** | | 0.0011 | 0.24324+-0.0360 | | 0.00079 | | 0.29730+-0.0451 | 0.00769 | | | 0.09009+-0.0271 | | 0.00101 | 0.27027+-0.0664 |
| **D3S1358** | 0.00691 | | 0.09009+-0.0136 | | 0.00068 | 0.29730+-0.0360 | | 0.00096 | | 0.25225+-0.0503 | 0.23296 | | | **0.00000+-0.0000** | | 0.00103 | 0.27928+-0.0497 |
| **THO1** | 0.09602 | | **0.00000+-0.0000** | | 0.00215 | 0.25225+-0.0521 | | 0.01722 | | 0.00901+-0.0091 | 0.01131 | | | 0.08108+-0.0252 | | 0.00021 | 0.35135+-0.0459 |
| **D13S317** | 0.02482 | | **0.00000+-0.0000** | | 0.00328 | 0.04505+-0.0203 | | 0.00732 | | 0.02703+-0.0139 | 0.02067 | | | 0.00901+-0.0091 | | 0.00435 | 0.09910+-0.0252 |
| **D16S539** | 0.02118 | | **0.00000+-0.0000** | | 0.00668 | 0.05405+-0.0201 | | 0.0421 | | **0.00000+-0.0000** | 0.01289 | | | 0.02703+-0.0139 | | 0.00448 | 0.11712+-0.0237 |
| **D2S1338** | 0.0213 | | **0.00000+-0.0000** | | 0.00132 | 0.26126+-0.0497 | | 0.02844 | | **0.00000+-0.0000** | 0.00441 | | | 0.17117+-0.0394 | | 0.00056 | 0.38739+-0.0385 |
| **D5S818** | 0.00582 | | 0.11712+-0.0360 | | 0.02471 | **0.00000+-0.0000** | | 0.00806 | | 0.01802+-0.0121 | -0.00254 | | | 0.44144+-0.0710 | | 0.00402 | 0.15315+-0.0333 |
| **FGA** | 0.00344 | | 0.07207+-0.0227 | | -0.00125 | 0.68468+-0.0364 | | 0.00288 | | 0.15315+-0.0360 | 0.00872 | | | 0.05405+-0.0201 | | 0.00109 | 0.27027+-0.0542 |
| **Bold values with significant p values (p value >0.003) of the studied population with other populations.** | | | | | | | | | | | | | | | | | |
|  |  | |  | |  |  | |  | |  | | |  |  | |  |  |
| **Table S3: Fst pairwise genetic distances between Teli population of Maharashtra and compared populations with their corresponding p-value** | | | | | | | | | | | | | | | | | |
| **Teli Population (Maharashtra) v/s** | | **Pooled populations belonging to geographical region of India** | | | | | **Population of Rajasthan** | | | | | **Mixed population of Maharashtra** | | | | | |
|  |  | **(India)** | | | | | **(Rajasthan)** | | | | | **(Maharashtra)** | | | | | |
|  |  | **Fst** | | **p-value** | | | **Fst** | | **p-value** | | | **Fst** | | | **p-value** | | |
| **D8S1179** | | 0.03643 | | **0.00000+-0.0000** | | | 0.0073 | | **0.00000+-0.0000** | | | -0.00293 | | | 0.86486+-0.0203 | | |
| **D21S11** | | -0.00039 | | 0.48649+-0.0411 | | | -0.00246 | | 0.88288+-0.0354 | | | -0.0041 | | | 0.98198+-0.0096 | | |
| **D7S820** | | -0.00229 | | 0.76577+-0.0205 | | | 0.00417 | | 0.08108+-0.0212 | | | -0.00418 | | | 0.92793+-0.0238 | | |
| **CSF1PO** | | 0.00002 | | 0.36036+-0.0525 | | | 0.00691 | | 0.02703+-0.0139 | | | -0.00287 | | | 0.68468+-0.0594 | | |
| **D19S433** | | 0.00497 | | 0.04505+-0.0152 | | | 0.00068 | | 0.26126+-0.0459 | | | -0.00224 | | | 0.75676+-0.0244 | | |
| **vWA** | | 0.00813 | | 0.00901+-0.0091 | | | 0.0101 | | 0.01802+-0.0121 | | | 0.00456 | | | 0.09910+-0.0212 | | |
| **TPOX** | | 0.00062 | | 0.34234+-0.0379 | | | 0.00447 | | 0.12613+-0.0242 | | | -0.00464 | | | 0.92793+-0.0274 | | |
| **D18S51** | | 0.10805 | | **0.00000+-0.0000** | | | -0.00038 | | 0.46847+-0.0379 | | | -0.00281 | | | 0.91892+-0.0184 | | |
| **D3S1358** | | 0.00076 | | 0.27027+-0.0507 | | | 0.00394 | | 0.10811+-0.0182 | | | -0.00225 | | | 0.72973+-0.0371 | | |
| **THO1** | | 0.00232 | | 0.25225+-0.0556 | | | 0.00259 | | 0.14414+-0.0473 | | | 0.00019 | | | 0.36937+-0.0417 | | |
| **D13S317** | | 0.00446 | | 0.07207+-0.0182 | | | 0.00429 | | 0.11712+-0.0273 | | | -0.00049 | | | 0.50450+-0.0639 | | |
| **D16S539** | | 0.00501 | | 0.06306+-0.0273 | | | 0.00591 | | 0.04505+-0.0203 | | | 0.00044 | | | 0.31532+-0.0339 | | |
| **D2S1338** | | -0.00023 | | 0.51351+-0.0622 | | | -0.00088 | | 0.54955+-0.0566 | | | -0.00274 | | | 0.88288+-0.0298 | | |
| **D5S818** | | 0.01091 | | 0.00901+-0.0091 | | | 0.0089 | | 0.01802+-0.0121 | | | -0.00334 | | | 0.81081+-0.0236 | | |
| **FGA** | | -0.00032 | | 0.45946+-0.0478 | | | 0.00143 | | 0.22523+-0.0389 | | | -0.00346 | | | 0.95495+-0.0151 | | |
| Bold values with significant p values (p value >0.003) of the studied population with other populations. | | | | | | | | | | | | | | | | | |
